# Case of Fracture of the Cranium

**Published:** 1852-09

**Authors:** H. J. Rice

**Affiliations:** Rockville, Indiana


*Case of Fracture of the Cranium.* By H. J. Rice, M. D., of Rockville, Indiana.

James Medley, aged fifteen years, sanguine temperament, in September 1851, received an extensive fracture of the cranium from the kick of a horse. The animal struck him with the front part of the shoe along the superciliary ridge of the right eye. The rim of bone forming the superior semicircular margin of the right eye came away by the slightest traction, this rim extending from one angular process to the other. The external wound through the common integuments and muscles extended transversely above the eye for the distance of three inches. Simple inspection and the use of a probe disclosed that nearly the whole superior portion of the frontal bone was driven far down on the brain.

The lines of fracture were three. The first commencing at the external angular process of the frontal bone, extended in a straight course towards the same process of the left side, passing the median line towards the left, an inch and a half, disclosing the superior margin of the frontal sinus. The second ranged along the temporal ridge till it met the coronal suture. The third passed transversely from the terminus of the second to a point an inch and a half to the left of the median line. The fourth, which would complete an irregular quadrilateral, I presume, was an example of bent bone, or a linear fracture of the external table, for this side was not depressed beyond the common level. Here was a shocking fracture; nearly the whole frontal region was driven down on the anterior lobes of the brain. The bone was firmly located in its false position, and the brain covered by the dura mater presented some hernial protrusions along the first line of fracture. My best endeavors to bring the plate to the common level failed. An incision through the muscles and integuments was made along the second line of fracture. A large flap could now be raised, for the depressed portion was perfectly stripped of its integuments; the pericranium being peeled from every point, and torn into shreds, was removed. It was still impossible to elevate, for the bone was firmly and almost immovably fixed in its false position. The internal table had scaled beyond the line of fracture in the external, at some points for the distance of quarter of an inch. A small trephine and Heys' saw were brought to bear, some snags of bone removed; and with elevators, by using cautiously what seemed to be a dangerous degree of force, the whole depressed portion was elevated to the common level. The dura mater, so far as could be detected, was not pierced, but was peeled from the depressed portion. The hæmorrhage was inconsiderable; a branch of the temporal artery required the ligature. Coagula were removed, the flap of tissues brought to its natural position, and confined by sutures, a sufficient aperture being left at the point from which the lines of fracture started, to give exit to the fluids of the wound. This extensive fracture and depression of bone did not produce corresponding psychological effects; there was a little mental hebetude.

*September 5th.* The patient was aware of all that transpired, answered questions, and knew his friends. There was partial insensibility to pain,—pulse 90 in the minute; skin moist. Charpie, saturated with tepid water, to be applied to the wound every half hour, was all the dressing required. Perfect rest and quietude enjoined, low diet, a saline purgative. R. Magnesiæ sulph. ℥i.

*Sept. 6th.* Passed a good night—bowels moved twice; pulse 100; skin moist; wound discharging and looks well; sutures removed, save one; union by the first intention in the line of incision; adhesive strips applied; rest and low diet.

*Sept. 7th.* Rested well during the night—pulse 100; no pain; some inflammation and redness of the flap; skin moist; healthy discharge from the wound. Ordered a saline cathartic.

*Evening of 7th.* Some pain in the head; irritability of stomach; restless; pulse 110; bowels not moved. Repeat the cathartic, magnesiæ sulph. ℥i.

*Sept. 8th.* Passed a restless night; face flushed; pulse 120; corded and tense; throbbing of temporal arteries; somewhat delirious; V. S. twenty-five ounces; magnesiæ sulph. ʒii. antim. tartrat. gr. $\frac{1}{4}$—to be taken every hour till the bowels move freely; after which the tr. emetic to be continued, gr. $\frac{1}{4}$ every hour.

*Sept. 9th.* Patient somewhat restless during the night—bowels freely moved; pulse 100; compressible; no irritation of stomach from the antimony. Continue the tr. emetic.

*Sept.* 10. Patient rested well during the night; skin moist; pulse 100; mind clear; healthy discharge from wound; low diet and rest.

*Sept. 11th.* Improving,—all symptoms of inflammatory action have subsided; lint saturated with warm water to be re-applied, (having been exchanged for cold applications during the period of excitement.) Nothing can be more injurious than heavy dressings to wounds of the cranium; the lighter the better for the patient.

It is unnecessary to give the history of the case any farther; from the application of simple and well established rules in the treatment of such cases, resulted a perfect cure.

In three weeks from the reception of this injury nothing but a cicatrix remained to tell the story of the accident.

Some ten months have elapsed since the injury—not a bad symptom has followed; sound as ever in body and mind, with a deformity by no means considerable.

I make this report, 1st, because it adds another recovery to the list of apparently hopeless cases.

2d. It proves that a cranial bone does not necessarily die when stripped of its dura mater and pericranial investments.

3d. As exhibiting the power of active depletion and tr. antimony in controlling the inflammatory process.

4th. As showing the vast amount of cerebral compression without corresponding manifestations over life and intellect.

